# Gender differences in health-seeking behaviour, diagnosis and treatment for TB

**DOI:** 10.5588/ijtld.21.0735

**Published:** 2022-06-01

**Authors:** S. Danarastri, K. E. Perry, Y. E. Hastomo, Kurniawati, K. Priyonugroho

**Affiliations:** 1 FHI 360 Indonesia Office, Jakarta, Indonesia; 2 FHI 360 Asia Pacific Regional Office, Bangkok, Thailand

Dear Editor, Gender influences routine TB diagnoses,^1^ treatment adherence^2^ and diagnostic delays,^3^ and informs how knowledge and perception affect TB patients and their health-seeking behaviours.^4^ Although gender influences the entire TB care continuum, its impact on TB has not been explicitly explored in Indonesia. This case study, part of the USAID Tuberculosis Private Sector Activity (USAID TBPS), investigated gender’s potential influence on Indonesia’s TB patient pathway and how a greater understanding of gender and health service delivery can improve access to care. We investigated gender-based patterns of TB care-seeking by conducting secondary analyses of the 2017 Indonesian Demographic and Health Survey (IDHS) and the National TB Surveillance Database (*Sistem Informasi Tuberkulosis Terpadu*, SITT) based on a previously published patient pathway analysis framework^5^ to find missing cases and align existing services to patients’ needs. The study was performed in six districts: Denpasar, Gresik, Medan, North Jakarta, Samarinda and South Jakarta (target geographies of USAID TBPS). Ethical approval was not required as no human subjects’ research was conducted.

Facilities identified from the two datasets were categorised into health system levels (0, 1, 2 and 3) and public vs. private facilities. Level 0 refers to community-based care; Level 1 to primary healthcare offering outpatient care delivered by health professionals; Level 2 to primary health facilities offering advanced inpatient and outpatient care with a wider range of diagnostic and treatment options; and Level 3 refers to specialised hospitals with advanced capabilities, including referral and teaching hospitals.^5^ χ^2^ tests were conducted to test statistical differences between variables. Most respondents (*n =* 49,627, 83%) of the IDHS (where we extrapolated data for initial care-seeking) were women, one-third of whom were 35–44 years of age. Within SITT data’s diagnostic phase, women comprised 45% (*n* = 6,518) of the 14,625 patients reported to have TB diagnostic access. Additionally, 74% (*n* = 10,847) of patients within SITT sought diagnostic care in public facilities, whereas 26% (*n* = 3,778) did so in private facilities (where 53% of patients were in Level 1 private and public facilities combined). Within SITT data’s treatment phase, women comprised 42% (*n* = 10,219) of the 24,648 patients reported to receive treatment. Additionally, 72% (*n* = 17,741) of all patients received treatment in public facilities, while 28% (*n* = 6,907) did in private facilities (where 48% of patients were in Level 1 private and public facilities combined) (Figure).

We observed a higher tendency for women to seek care in private primary clinics compared to men, who opted for Level 0 pharmacy visits: 33% (*n* = 16,377) of women sought care at Level 1 private facilities, whereas 30% (*n* = 3,002) of men sought care at Level 0 private facilities. As demonstrated by a study in Indonesia, 73% of Level 1 public facilities, 73% of Level 2 public facilities and 59% of Level 2 private facilities have adequate diagnostic capacity.^6^ However, diagnostic capacity was found in only 2% of Level 1 private facilities,^7^ where our data suggest that 33% of women (*n* = 16,377) and 29% of men (*n* = 2,902) initially sought care. Likewise, for 32% of women (*n* = 15,881) and 30% of men (*n* = 3,002) who preferred Level 0 private facilities for initial care-seeking in our sample, diagnosis is likely to be unavailable. Thus, for those who initially sought care at private Levels 0 and 1, 65% of women (*n*=32,258) and 59% of men (*n* = 5,905) were unlikely to be diagnosed at their first point of care.

At the point of diagnosis, our data suggest a higher proportion of patients accessed care in public facilities than in private facilities – at 75% (*n* = 6,076) and 73% (*n* = 4,771) for men and women, respectively. These findings align with a previous study regarding the location of diagnostic equipment.^6^ However, when disaggregating data by gender, women were more likely to access diagnosis in Level 2 and 3 private facilities than men, making Indonesian women more likely to experience delays in receiving TB diagnosis and less likely to be offered a sputum test for bacteriological confirmation. This is because private facilities have reduced diagnostic capabilities compared to the public sector.^8^,^9^ Improving linkages between public (where diagnostic tools are primarily located) and private sectors (where patients primarily visit) for diagnostic access will allow patients to remain at preferred points of care. Such integration has been implemented through the public-private mix (PPM) initiative in Indonesia. However, despite efforts to link private facilities to the National TB Control Programme (NTP) and an increase in PPM engagement, the number of private facilities included remains low, especially at the primary care level. Furthermore, despite private facility PPM engagement, notification rates are suboptimal, with estimates of high underreporting at Levels 1 and 2. More patients accessed the public than the private sector for treatment (men: *n* = 10,492, 73%; women: *n* = 7,249, 71%). When disaggregated by level, a greater percentage of men and women were treated at Level 1 public facilities. The NTP is heavily dependent on public facilities. Patients accessing care at private facilities may suffer from out-of-pocket expenditure for TB drugs unless their facility is engaged in the NTP’s PPM programme, potentially deterring patients from receiving care at private facilities or leading to treatment discontinuation. Furthermore, the national health insurance scheme mandates that uncomplicated TB cases should be treated within Level 1. Although this mandate does not exclude Level 1 private facilities, our study found that most TB cases were referred to public facilities. Expanding treatment access to private facilities could reduce treatment transfers and allow patients to remain at preferred points of care. To note, men and women over 65 were more likely to seek treatment at Level 2 facilities (with the proportion of women higher than men, *P* < 0.001), suggesting that engaging Level 2 facilities to systematically search for TB among older people may increase case-finding. Furthermore, integrating TB services with geriatric clinics within Level 1 may provide added value of care for older people and improve coverage.

**Figure:**
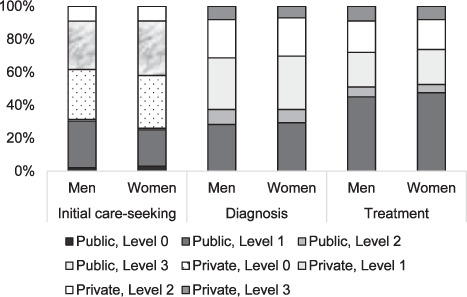
Patient location along the TB continuum of care disaggregated by gender within various levels in the public and private sector, from initial care-seeking to diagnosis and treatment points of care, along the TB care continuum. The figure demonstrates the different health facilities accessed by TB patients at three points of care: initial care, diagnosis and treatment. Most initial care-seeking occurred at private sector Level 0 (community-based care), where 30% of men and 32% of women sought care (extrapolated from IDHS data). Most diagnoses occurred at Level 3 public facilities, with 31% patients (men and women; extrapolated from SITT TB.06 data). Treatment primarily occurred at public sector Level 1 (primary outpatient care), including 45% of all patients (men and women; extrapolated from SITT TB.03 data). IDHS = Indonesian Demographic and Health Survey; SITT = Sistem Informasi Tuberkulosis Terpadu (integrated tuberculosis information system).

Indonesia aims to successfully treat at least 90% of all cases to reach the End TB goal of TB elimination by 2030.^10^ Our findings demonstrate at least an 80% treatment success rate, although women were more likely to complete treatment than men (*P* < 0.001). Providing treatment adherence support to male patients could improve treatment success.

Our case study has several limitations. Data were drawn from six districts, which may not be representative of Indonesia, nor generalizable. Our findings (generated via a proxy) operated on assumptions of TB service availability, which may not reflect actual conditions. Moreover, our analysis was limited to understanding general care-seeking patterns and was unable to deduce reasons for patients’ shifting behaviour along the care continuum. Despite these limitations, the study provides preliminary insights into gender differences along the TB care continuum.
